# Insulin: Trigger and Target of Renal Functions

**DOI:** 10.3389/fcell.2020.00519

**Published:** 2020-07-29

**Authors:** Ana F. Pina, Diego O. Borges, Maria João Meneses, Patrícia Branco, Rita Birne, Antonio Vilasi, Maria Paula Macedo

**Affiliations:** ^1^Centro de Estudos de Doenças Crónicas, NOVA Medical School/Faculdade de Ciências Médicas, Universidade Nova de Lisboa, Lisbon, Portugal; ^2^ProRegeM Ph.D. Programme, NOVA Medical School/Faculdade de Ciências Médicas, Universidade Nova de Lisboa, Lisbon, Portugal; ^3^Department of Medical Sciences, University of Aveiro, Aveiro, Portugal; ^4^Molecular Biosciences Ph.D. Programme, Instituto de Tecnologia Química e Biológica António Xavier, Universidade Nova de Lisboa, Oeiras, Portugal; ^5^Department of Nephrology, Centro Hospitalar Lisboa Ocidental, Lisbon, Portugal; ^6^Portuguese Diabetes Association – Education and Research Center (APDP-ERC), Lisbon, Portugal; ^7^Institute of Clinical Physiology – National Research Council, Reggio Calabria Unit1, Reggio Calabria, Italy

**Keywords:** diabetic nephropathy, insulin, insulin resistance, insulin clearance, albuminuria

## Abstract

Kidney function in metabolism is often underestimated. Although the word “clearance” is associated to “degradation”, at nephron level, proper balance between what is truly degraded and what is redirected to *de novo* utilization is crucial for the maintenance of electrolytic and acid–basic balance and energy conservation. Insulin is probably one of the best examples of how diverse and heterogeneous kidney response can be. Kidney has a primary role in the degradation of insulin released in the bloodstream, but it is also incredibly susceptible to insulin action throughout the nephron. Fluctuations in insulin levels during fast and fed state add another layer of complexity in the understanding of kidney fine-tuning. This review aims at revisiting renal insulin actions and clearance and to address the association of kidney dysmetabolism with hyperinsulinemia and insulin resistance, both highly prevalent phenomena in modern society.

## Introduction

Insulin is a vital hormone with several functions among which its central role in energy and glucose homeostasis is fundamental (Plum, 2006). Beyond the traditional crosstalk between the most recognized insulin-responsive organs (liver, adipose tissue, and skeletal muscle), kidney insulin action and resistance have recently been suggested as critical components of metabolic and dysmetabolic states (Artunc et al., 2016). The important role of insulin in the kidney is further corroborated by the increased prevalence of chronic kidney disease in subjects with type 2 diabetes mellitus (T2DM) and non-alcoholic fatty liver disease (NAFLD; Musso et al., 2014; Kim et al., 2018; Mok et al., 2019; Kiapidou et al., 2020). Furthermore, the kidney can adjust the levels of essential players of nutrient homeostasis such as glucose and insulin (Krebs, 1963; Chamberlain and Stimmler, 1967).

First reports of insulin actions in the kidney were originated in the 1950s (Farber et al., 1951). Since then, the knowledge acquired has dramatically increased, nonetheless much remains unveiled. It is now known that insulin impacts on tubular glucose reabsorption (Nakamura et al., 2015). In fact, a whole therapeutic class based on this mechanism was recently developed. Additionally, hyperinsulinemia driven by pancreatic hypersecretion and/or impairments in hepatic insulin clearance could explain changes in glomerular filtration rates and increased renal gluconeogenesis, which in turn causes fatty liver deposition and, as a direct consequence, may lead to dysglycemia (Bril et al., 2014; Naderpoor et al., 2017; Jung et al., 2018; Bergman et al., 2019).

Kidney is, in fact, the most critical organ responsible for insulin clearance after the liver (Elgee and Williams, 1954; Narahara et al., 1958); still, its relevance in the maintenance of proper insulinemia and insulin sensitivity is underestimated. The control of insulin clearance/action exerted by the kidney is complex. Furthermore, the diversity of insulin actions is only possible thanks to the differentiation of specific kidney segments in which insulin regulates different pathways. In this review, we will briefly describe insulin production and its main actions in the liver, adipose tissue, and skeletal muscle. Moreover, we will focus on kidney and on renal insulin actions as well as insulin clearance/degradation. Overall, this work aims at highlighting the critical role of kidney-insulin interplay in the development of dysmetabolism.

## Overview of Insulin Biosynthesis and Non-Renal Action and Metabolism

The insulin gene (*INS*) is represented by only one copy in the human genome, and its transcription is mainly regulated by the same enhancers of other glucose-related genes (Andrali et al., 2008; Gao et al., 2014). In pancreas β-cell, *INS* is first transcribed as preproinsulin and, after cleavage and folding in the rough endoplasmic reticulum (ER), proinsulin is formed (Hutton, 1994). The conversion into insulin occurs after the removal of the c-peptide in the Golgi apparatus, with a final maturation achieved by c-terminal amino acid elimination (Steiner, 2004). Mature insulin is composed of A and B chains linked by two disulfide bonds, with a third bond within A chain (Mayer et al., 2007). For storage, insulin is complexed in hexamers with zinc, and an actin network is involved in the organization of mature insulin granules for first and second insulin secretion phases (Kalwat and Thurmond, 2013).

Insulin secretion is triggered by cytoplasmic increments in ATP levels, derived from glucose internalization and subsequent metabolism (Prentki and Matschinsky, 1987). This increase in ATP shuts down membrane ATP-sensitive K^+^ channels, depolarizing plasma membrane, and activating voltage-gated calcium channels (Detimary et al., 1998). Indeed, the resultant Ca^2+^ influx seems to be necessary for the fusion of insulin granules with the plasma membrane, ending up in its secretion (Hou et al., 2009). The first wave of insulin secretion is composed of primed granules that are in close proximity with plasma membrane (Olofsson et al., 2002). However, most of these granules remain more distant from the plasma membrane and are further boosted with newly synthesized insulin that will culminate in 75–95% of total insulin secretion in the second phase (Kou et al., 2014).

With the insulin pools ready to be released, insulin secretion does not occur in a constant manner, but in waves of 4–5 min interval (Song et al., 2000). The pulsatile secretion profile is considered primordial for proper insulin responsiveness, as constant release would result in down-regulation of insulin receptor (INSR) expression (Matveyenko et al., 2012). After binding and activation of its receptor, insulin, as an anabolic hormone, promotes increased biosynthesis of macromolecules further needed to maintain energetic balance during nutrient privation (Bedinger and Adams, 2015).

**FIGURE 1:**
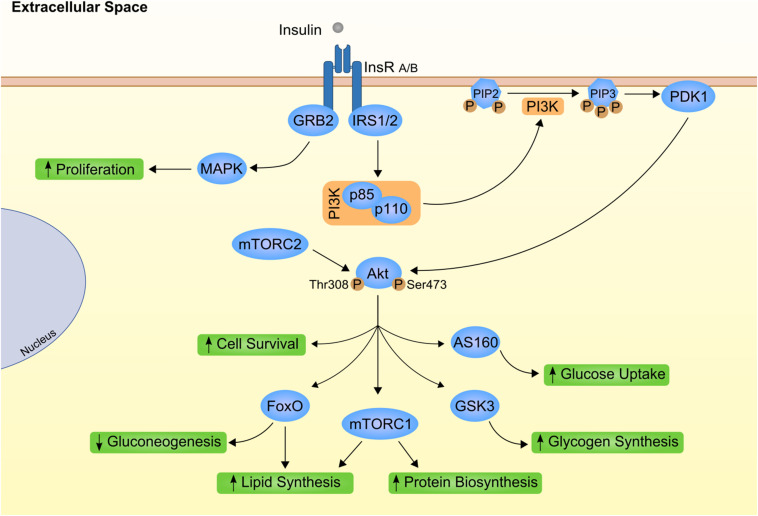
Canonical insulin signaling cascade. Insulin (gray circle) binds to its transmembrane kinase receptor at cell membrane and triggers insulin signaling cascade. Insulin receptor present two isoforms with distinct affinity for insulin already associated to differential insulin internalization (INSR A and INSR B; Calzi et al., 1997). Binding of insulin auto-phosphorylates INSR at Tyr-960. Further recruitment and phosphorylation of insulin receptor substrate (IRS) 1 and 2 will mostly result in subsequent activation of phosphoinositide-dependent kinase 1 (PDK1) through phosphoinositide-3 kinase (PI3K; Yamada et al., 2002). PDK1 is responsible for propagation of insulin signal to one of the most important downstream effectors, Akt. Importantly, Akt has two distinct phosphorylation sites, Thr308 activated by PDK1 (Alessi et al., 1997) and Ser473 phosphorylated by mammalian target of rapamycin complex (mTORC) 2 protein (Bayascas and Alessi, 2005). Finally, fully activated Akt can interact with different proteins, eliciting different effects as stimulation of glucose uptake and glycogen synthesis by AS160 and glycogen synthase kinase 3 (GSK3), respectively (Ng et al., 2008). On the other hand, INRS activation also promote growth factor receptor-bound protein 2 (GRB2) interaction with Shc proteins and activation of mitogen activated protein kinases (MAPK; Skolnik et al., 1993; Xu et al., 2006). This as part of the insulin-mediated proliferative stimuli. PIP2, phosphatidylinositol 4,5 biphosphate; PIP3, phosphatidylinositol 3,4,5 triphosphate.

At cellular level, insulin mostly relies on the specific bind to its transmembrane receptor to perform its actions (Figure 1). Insulin receptor is a tyrosine kinase receptor with two extracellular α subunits and two transmembrane β subunits (Ye et al., 2017). Insulin binding to INSR produces an autophosphorylation that leads to the internalization of the complex and activation of downstream effectors. INSR substrate 1 (IRS1) and 2 (IRS2) are the main targets that intracellularly coordinate insulin action (Thirone et al., 2006; Kubota et al., 2016). In the liver, the activity of these proteins is differently controlled by insulin. Although both IRS1 and IRS2 are degraded after insulin activation, only IRS2 is transcriptionally repressed by insulin activation (Hirashima et al., 2003). Further transduction of insulin signaling occurs via phosphatidylinositol 3-kinase (PI3K)/AKT phosphorylation. AKT or Protein kinase B (PKB) are considered the main effectors of metabolic insulin actions (Taniguchi et al., 2006; Molinaro et al., 2019). On the other hand, AKT1 and 3 are the leading players of mitogenic effects of insulin by RAS/mitogen activated protein kinase (MAPK) cascade and crosstalk with growth hormones (Xu et al., 2006; Xu and Messina, 2009; Qiu et al., 2017).

Given the scope of this review, we will summarize insulin actions on non-renal target organs, but detailed information is available in recent reviews (Gancheva et al., 2018; Tokarz et al., 2018). The first organ to be reached by insulin is the liver, where 50–70% is removed at first pass (Duckworth et al., 1998). In hepatocytes, insulin signaling recruits forkhead box transcription factor 1 (FoxO1) to the cytoplasm, inhibiting the transcription of gluconeogenic enzymes (Ling et al., 2018). In such an abundance of glucose, insulin also stimulates the synthesis of glycogen by phosphorylation of glycogen synthase kinase 3 (GSK3), which leads to dephosphorylation and activation of glycogen synthase (McManus et al., 2005). After the replenishment of liver glycogen stores in the fed state, glucose starts to be degraded to pyruvate, which is the precursor of lipid biosynthesis via *de novo* lipogenesis (DNL; Sanders and Griffin, 2016). Herein, insulin signaling is also crucial for upregulation of sterol regulatory element binding protein 1c (SREPB1c), a transcription factor associated to further transcription of DNL (Oh et al., 2003; Roder et al., 2007; Krycer et al., 2010).

Only a fraction of the insulin that reaches the liver will get to the periphery, to perform its action at skeletal muscle, adipose tissue, and other insulin-sensitive organs. Skeletal muscle is the greater insulin-dependent “kidnapper” of glucose, it is responsible for ∼60 to 80% of whole-body glucose disposal (DeFronzo et al., 1976; Fernandes et al., 2011). Insulin signals to myocytes through classical signaling, promoting glucose uptake by the dynamic translocation of glucose transporter 4 (GLUT4; Zorzano et al., 1996). Glucose is here mainly used for glycolysis or stored as glycogen as already described for the liver.

During the fed state, a clear inhibitory effect in lipolysis and consequent blunt of free fatty acid (FFA) release is mediated by insulin acting on adipocytes (Dimitriadis et al., 2011). The resultant decrease in FFA availability reduces its hepatic uptake and fatty acid oxidation, changing the disposal of substrates that are mostly used for gluconeogenesis (Galgani et al., 2008). Not exclusively for liver, the effect of insulin in reducing adipose tissue FFA release is important for other organs that, at fed state, change energy source to glucose and recover glycogen stores contributing to normoglycemia (Petersen and Shulman, 2018). In this context, glucose uptake is also stimulated by insulin in the fed state at adipocytes. Furthermore, translocation of glucose transporters to the membrane of adipocytes was very well characterized in 3T3-L1 adipocytes by activation of the canonical PI3K/AKT2 pathway (Ng et al., 2008).

As mentioned above, all these organs expressing INSR are responsible for insulin clearance and local degradation. However, a significant part of insulin degradation is played in the liver (50–70%) by its specialized machinery of internalization and degradation (Najjar and Perdomo, 2019). In the case of internalization, carcinoembryonic antigen-related cell adhesion molecule 1 (CEACAM-1) phosphorylation by insulin promotes rapidly receptor-mediated uptake (Poy et al., 2002). Carcinoembryonic antigen-related cell adhesion molecule 1 expression occurs preferentially in liver, and restoration of its function only at liver of null CEACAM-1 mice reverse the hyperinsulinemia and fatty liver deposition observed in global knockout CEACAM-1 model (Russo et al., 2017). Regarding degradation, insulin-degrading enzyme (IDE) is considered the most critical protease to cleave insulin intracellularly and is highly expressed in the liver (Kuo et al., 1993).

The amount of insulin that remains in circulation after liver first pass (30–50%) will be mostly degraded by the kidney (Elgee and Williams, 1954; Narahara et al., 1958). However, the kidney is not just an endpoint for insulin degradation, as insulin also acts throughout the entire nephron. Whereas hepatic insulin clearance is regaining attention as the very first alteration leading to hyperinsulinemia, compensatory mechanisms that could emerge from the kidney are not well known. Impairments in hepatic insulin clearance increase peripheral insulinemia, and very little is described regarding the exact effects of the increased amount of insulin that will further reach the kidney.

## Kidney and Insulin Actions

**FIGURE 2:**
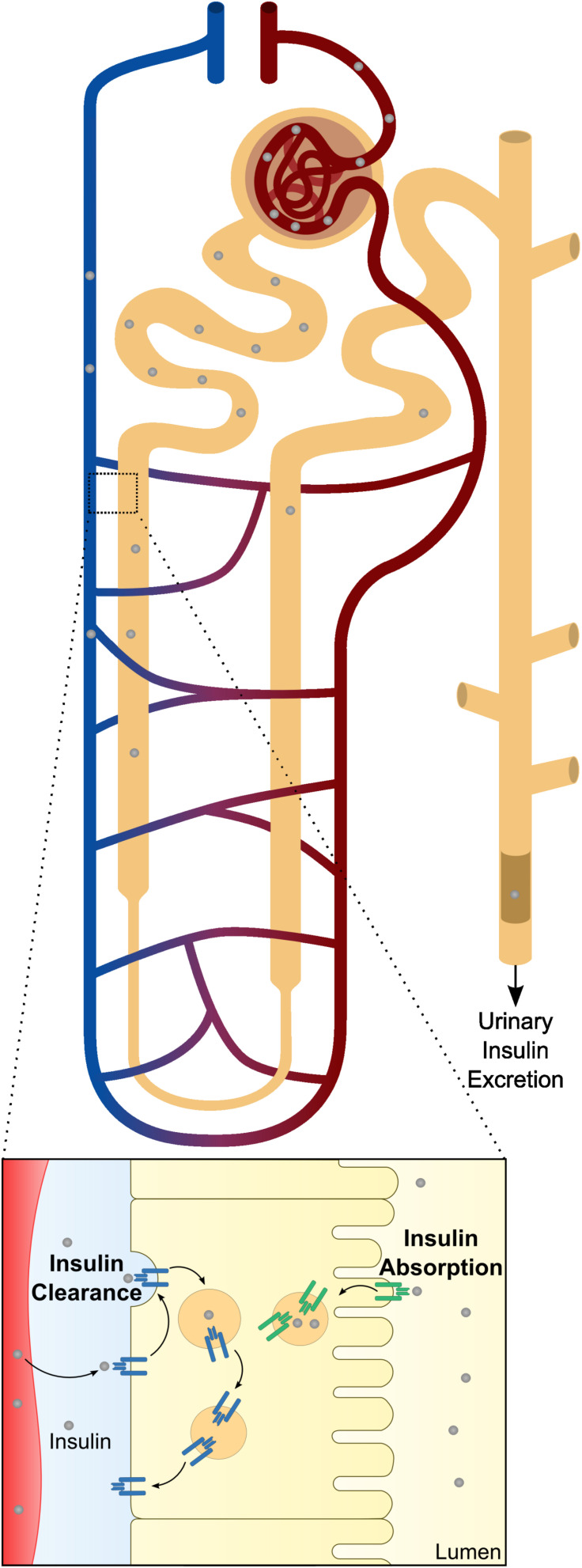
Schematic representation of renal insulin handling. Insulin is depicted in gray circles at different portions of the nephron. As insulin is a small molecule, it will be fully filtered by the glomerular system until it reaches the proximal tubule. At proximal tubule cells, almost all the filtered insulin will be absorbed at the luminal membrane. In physiological conditions, only a small percentage will be excreted in urine. Beyond glomerular filtration, insulin also raise from the perivenous capillaries. In a closer look to the proximal tubule cells is represented both mechanisms of insulin clearance. The increased levels of insulin receptor (INSR) and consequent increased uptake of insulin at the basolateral membrane is also depicted.

Kidney works together with the liver for maintenance of optimal insulin levels. In humans, kidney removes 6–8 U of insulin per day by two major routes (Figure 2), that apparently have distinct preferential physiological goals: post-glomerular secretion (insulin signaling) and glomerular filtration (nutrient conservation and homeostasis) (Ritz et al., 2013). In the kidney, insulin acts at multiple sites along the nephron, from the glomerulus (Coward et al., 2005; Welsh et al., 2010; Mima et al., 2011; Lay and Coward, 2014) to the renal tubule (Tiwari et al., 2008, 2013; Li et al., 2013), to modulate different functions such as glomerular filtration, gluconeogenesis, renal sodium handling, among others.

Many alterations in insulin associated mechanisms in the kidney are driven by insulin resistance (IR), leading to diabetic nephropathy if not reestablished (Svensson and Eriksson, 2006). In the Asiatic population, it was described that the prevalence of diabetic nephropathy with impaired estimated glomerular filtration rate (eGFR) (with or without albuminuria) was 31.6%, and the prevalence of albuminuria (with or without impaired eGFR) was 16.9 and 22.0%, respectively (Mok et al., 2019). Patients may already show diabetic kidney disease (DKD) at the time of T2DM diagnosis. Nevertheless, 10 years after the diagnosis of T2DM, low-level albuminuria is present in 24.9% of the patients and 5.3% already present macroalbuminuria (Adler et al., 2003).

### Glomerular Insulin Actions

**FIGURE 3:**
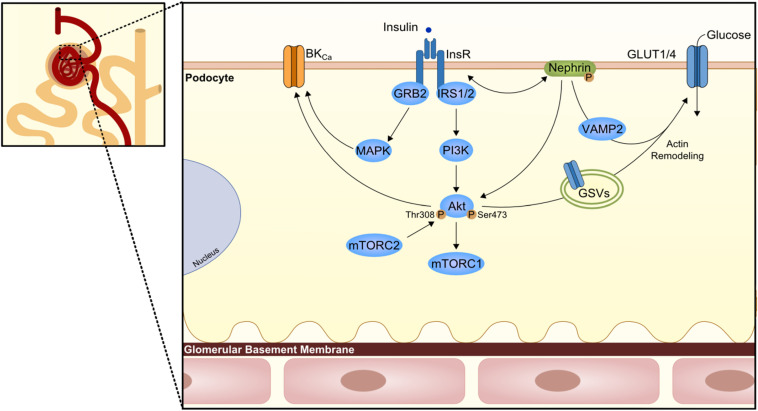
Podocyte insulin signaling. Podocytes are the first cells to interact with insulin at the nephron and express several proteins of the canonical insulin signaling pathway. However, here the podocyte-specific protein nephrin is known to have a role in the trafficking of glucose transporters (GLUT1 or GLUT4) to podocyte membrane and consequently promote glucose uptake. The trafficking seems to involve Vamp2 and actin remodeling. On the other branch of insulin signaling, an effect in the large-conductance Ca^2+^-activated K^+^ channels (BK_*Ca*_) is also important for maintenance of podocyte integrity and proper glomerular filtration. GRB2, growth factor receptor-bound protein 2; GSV, GLUT storage vesicle; VAMP2, Vesicle-associated membrane protein 2.

Podocytes are the primary constituent cell of the glomerulus, with their long finger-like projections to the glomerular capillaries at the glomerular basement membrane (GBM). These cells have intercellular junctions that form filtration barriers to help maintaining normal renal function. When damaged, podocytes lose their arrangement resulting in a reduction in barrier function. Indeed, one of the DKD features is the podocyte loss with consequent albuminuria (Pagtalunan et al., 1997; Wolf et al., 2005). Lately, it has been unveiled the role of insulin in podocytes (Figure 3); in other words, podocyte IR gives rise to the albuminuric feature.

Podocytes reveal to express proteins of the insulin signaling canonical pathway, namely the INSR, and both IRS1 and IRS2 (Coward et al., 2005; Santamaria et al., 2015), with IRS2 being the most prevalent one. Coward et al. (2005) depicted the effect of insulin in human podocytes, which results in glucose uptake not only through GLUT4 but also through glucose transporter 1 (GLUT1; Figure 3). As insulin promotes the translocation of GLUT4 to the membrane through the activation of PI3K-AKT2-PkB pathway, there is a remodeling of the cortical actin of the cytoskeleton with subsequent contraction (Welsh et al., 2010). In compliance, podocytes-specific deletion of INSR in mice revealed DKD features based on substantial albuminuria and histological features as podocyte foot structure loss and glomerulosclerosis (Welsh et al., 2010). AKT seems to play a central role as its phosphorylation appears to be severely affected in several models of both type 1 diabetes mellitus (T1DM; streptozotocin-induced T1DM) and T2DM (db/db mice, Zucker rats) (Tejada et al., 2008; Mima et al., 2011). Moreover, AKT isoform 2 deletion results in serious glomerular lesions in mice. This can lead to rapid disease progression, also associated with tubular dilatation and microalbuminuria (Canaud et al., 2013). Other relevant players that might contribute to podocyte IR is SH2-domain-containing inositol phosphatase 2 (SHIP2), a down regulator of the PI3K signaling pathway shown to be upregulated in the Zucker rats. Moreover, protein tyrosine-phosphatase 1B (PTP1B), a negative regulator of the INSR activity, or phosphatase and tensin homolog when increased, appears to also compromise the insulin signaling pathway (Mima et al., 2011; Garner et al., 2018).

Podocytes also present an insulin-dependent alternative pathway, the cyclic guanosine monophosphate(cGMP)-dependent protein kinase G (PKG), from which the PKG isoform I-alpha levels are increased in glomeruli of the hyperinsulinemic Zucker rats (Piwkowska et al., 2013). These high insulin levels increase glomerular barrier albumin permeability through a PKGI-reliant mechanism via the NAD(P)H-dependent generation of superoxide anion.

An important player in podocytes physiology is the protein nephrin, a podocyte-specific protein, which is responsible for the maintenance of the integrity of the filtration barrier. In fact, nephrin mutations are involved in severe nephrotic syndrome (Lenkkeri et al., 1999). Nephrin appears to play a most outstanding role in the trafficking of GLUT4 and GLUT1 by interacting with Vamp2 as well as by interacting with insulin-stimulated actin remodeling (Coward et al., 2007; Lay et al., 2017). Of interest, nephrin can also induce phosphorylation of p70S6K in a PI3K-dependent manner independently of INSR/AKT2 activation (Villarreal et al., 2016; Figure 3).

The development of glomerular IR is triggered by several factors: high glucose and/or insulin levels, increased FFAs levels, or an inflammatory milieu. Moreover, insulin signaling appears to be relevant for the adaptative ER stress response in DKD; this is the case for mice with podocyte-specific heterozygous INSR deletion (Madhusudhan et al., 2015). In support of this view, stable overexpression of INSR or knock-down of PTP1B was protective against ER stress (Garner et al., 2018). Podocyte mitochondria play an essential role in cellular metabolism. When dysfunctional as a result of reactive oxygen species production, mitochondria triggers apoptosis, which can also be observed along with a compromised IR state (Susztak et al., 2006). Certainly, the preservation or reestablishment of podocyte integrity is essential in the prevention of the onset and development of DKD.

## Insulin Tubular Actions

In the kidney tubule, insulin has several roles: metabolism, electrolyte and acid-base regulation and absorption of filtered substances. However, the exact mechanisms by which insulin performs these distinct roles is not fully understood. Nonetheless, it seems that, at least some of them, are mediated by INSR, and can be explained by the recruitment of specific IRS, as recently shown by Nakamura et al. (2020) specifically for gluconeogenesis and sodium reabsorption regulation. Still, there are overlapping mediators in downstream pathways. In the following paragraphs we will summarize the most relevant and well-known insulin actions in the tubular segment.

**FIGURE 4:**
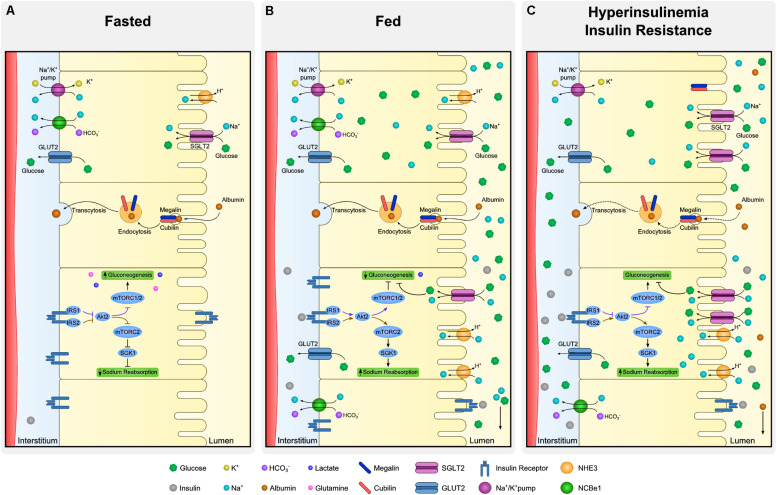
Dynamics of proximal tubule cells at fasting, fed and insulin resistant states. Proximal tubule cells are subjected to distinct microenvironments (lumen and interstitium) and the regulation of absorption and reabsorption of molecules is complex. Although all the described processes occur in every cell of the proximal tubule simultaneously, each specific process is illustrated in a different cell. At fasting **(A)**, low levels of insulin allow expression of gluconeogenic enzymes whereas sodium reabsorption is downregulated. Expression of glucose transporter 2 (GLUT2) at basolateral membrane is mostly associated to glucose output and not to its uptake. Moreover, albumin absorption is performed by megalin and cubilin at luminal membrane and transcytosis allow albumin to be rerouted back to the organism. At fed state **(B)**, increased availability of insulin and glucose promote drastic changes in proximal tubule dynamics. In the case of insulin, luminal uptake is mostly associated to degradation and basolateral to signaling activation. Insulin receptor (INSR) activation downregulates gluconeogenesis and increases sodium reabsorption by different proteins as type 3 Na-H exchanger (NHE-3) and sodium-glucose transport protein 2 (SGLT2). Together with sodium, SGLT2 also co-transport glucose from the lumen. Finally, hyperinsulinemia is linked to perturbations of proximal tubule cells in many aspects **(C)**. As in many other organs, insulin signaling desensitization is associated to inefficient inhibition of gluconeogenesis contributing to maintenance of increased levels of glucose. Derangements at podocyte level increases filtration of albumin and overloads luminal capacity of reabsorption. Such impairment in albumin reabsorption culminates with albuminuria, frequent observed in hyperinsulinemic states.

Insulin receptor is present throughout the entire nephron (Butlen et al., 1988; Stechi et al., 1994), however, insulin binding capacity of luminal and basolateral membrane tubule cells is different. There is evidence showing same affinity of INSR in both membrane sides of the cell, nonetheless its abundance is asymmetrical (Hammerman, 1985). In fact, the binding capacity of the contraluminal compared to luminal membrane seems to be several times greater due to higher expression of INSR (Talor et al., 1982). Figure 4 summarizes insulin signaling in proximal tubule (PT), regarding its actions in both gluconeogenesis and sodium reabsorption. Additionally, insulin actions through INSR are thought to be different in the proximal and distal nephron regions. Tiwari et al. (2013) demonstrated, in a mouse model with deletion of INSR in the kidney tubule cells, that depending on the segment targeted with INSR deletion, there were different phenotypes further described. In case of decreased INSR at PT, animals had a mild diabetic phenotype, without increased IR when compared to control. These animals shown to have an higher activity of gluconeogenesis enzymes (Tiwari et al., 2013). On the other hand, in animals with the deletion of INSR targeted to distal parts of the tubule, elevated blood pressure and impaired sodium excretion was observed (Tiwari et al., 2008).

### Glucose Reabsorption

Glucose is reabsorbed by the PT cells from the kidney tubule lumen to the bloodstream (Figure 4A). In the kidney, GLUT2 is in the basolateral membrane and diffuses glucose out of the cell, contrary to the liver, where GLUT2 acts in glucose uptake. Sodium-glucose transporter proteins (SGLT) are responsible for glucose and sodium co-transport by the luminal membrane of kidney cells. Sodium-glucose co-transporter 2 (SGLT2) is a high-capacity/low affinity sodium-glucose cotransporter present in the apical membrane of proximal convoluted tubule cells (Vallon et al., 2011). These transporters are responsible for approximately 90% of filtered glucose reabsorption, and an important target for T2DM therapy (SGLT2 inhibitors; DeFronzo et al., 2012). The remaining 10% of glucose in the tubule is absorbed by SGLT1, a low capacity and high affinity sodium-glucose cotransporter (Wright et al., 2011). Kidney has a threshold for glucose excretion which, in healthy conditions, relates to a glycemic value around 180 mg/dl (Figure 4B). This threshold, however, can be altered in diabetes (Rave et al., 2006). It is not clear if SGLT2 glucose transport is or not directly dependent on insulin signaling (Ferrannini et al., 2020). Nonetheless, SGLT2 expression was shown to be upregulated by insulin on human cultured PT cells, in a dose-dependent manner (Nakamura et al., 2015). Therefore, in hyperinsulinemic states, frequently observed in prediabetes and T2DM, an excessive glucose absorption can be observed. Of notice, glucose is still highly absorbed by SGLT2 in IR states, suggesting that this mechanism is not affected by IR, though it is upregulated by hyperinsulinemia. In this case, a vicious cycle can happen where increased insulin levels drive an increase in glucose SGLT2 overexpression increasing glycemic levels which in turn will increment the insulin secretion (Figure 4C). Therefore, SGLT2 overexpression inhibition is a potential new target for highly prevalent hyperinsulinemia related conditions, namely some dysglycemic phenotypes and obesity. In this case, lowering insulin levels could be paramount to prevent SGLT2 overexpression. Thus, SGLT2 inhibitors might become a relevant therapeutic approach for hyperinsulinemia related conditions, other than T2DM, namely obesity and prediabetes.

### Gluconeogenesis Regulation

Kidney has a major role in gluconeogenesis along with the liver and the intestine. Gluconeogenesis occurs mainly in PT cells essentially from lactate and glutamine (Figure 4A). Moreover, PT cells do not use glucose, as they get their energy mostly from fatty acid oxidation (Gerich et al., 1963). Insulin regulates gluconeogenesis in PT cells to meet the fluctuating needs of the body. While in the fasting state the kidney contributes in 40% to overall gluconeogenesis, in the post-absorptive state its contribution drops to 20% (Gerich et al., 1963; Figure 4B).

Nakamura et al. (2020) demonstrated that insulin directly inhibits gluconeogenesis in isolated PT through IRS1/AKT2/mTORC1/2, and that mTORC1 positively regulates insulin signaling (Nakamura et al., 2020). Specifically, in the fasting state, suppressed insulin signaling increases FoxO1 activity, increasing the expression of gluconeogenic genes, such as PEPCK and glucose-6-phosphatase. In parallel, decreased glucose reabsorption via SGLT2 on the luminal membrane downregulates the NADH/NAD^+^ ratio, activating sirtuin 1 and peroxisome proliferator-activated receptor-gamma coactivator 1α (PGC1α), a coactivator of FoxO1. Therefore, these two mechanisms lead to enhanced gluconeogenesis. On the contrary, in the fed state, the increased insulin levels and glucose reabsorption result in the suppression of gluconeogenesis through downregulation of the previously mentioned gluconeogenic genes (Nakamura et al., 2015; Sasaki et al., 2017). Whereas glucose is reabsorbed by the luminal membrane, insulin interacts with the basolateral membrane, thus gluconeogenesis regulation results from the integration of signals from distinct cell microenvironment.

### Sodium

Insulin impacts on the fine-tuning of several electrolytes by the kidney. Among them sodium handling is probably the best described (DeFronzo et al., 1975). However, the insulin role on sodium retention in normoglycemia is not completely established. Regulation of sodium absorption is paramount to maintain the extracellular volume in a physiological range. Kidney is the principal organ involved in sodium excretion, responding and adapting to sodium intake (Krekels et al., 2015). Additionally, sodium has a major role in driving electrochemical forces that support kidney primary role in fine-tuning body composition. Sodium reabsorption and excretion results from the integration of a complex network of sensors, neural-hormonal stimuli and hemodynamic and metabolic mechanisms (Frame and Wainford, 2017).

Contrary to glucose, sodium is absorbed along the nephron by distinct apical sodium transport proteins. Usually, approximately 65% of filtered sodium is reabsorbed in the PT along with water, mainly through type 3 Na-H exchanger (NHE-3) and SGLT proteins. In addition, the thick ascending limb is responsible for approximately 25% of reabsorption through sodium-potassium chloride cotransporter-2 (NKCC2). Finally, 5–10% of sodium is reabsorbed in the collecting duct by epithelial sodium channel (ENaC) and less than 10% is excreted in urine (Esteva-Font et al., 2012).

The association of insulin with sodium absorption was suggested almost a century ago (Atchley et al., 1933), nonetheless discrimination of insulin- and glucose-mediated effects has not been clarified (DeFronzo et al., 1976; Manhiani et al., 2011). It is still under debate if insulin has a causal effect on hypertension under normoglycemia. Nevertheless, it is known that insulin stimulates sodium absorption in all the tubule segments where it takes place (Kirchner, 1988; Friedberg et al., 1991; Ghezzi and Wright, 2012). In the PT, insulin regulates several sodium transporters, in both luminal (NHE-3, SGLT2) and basolateral membrane (Na/K-ATPase, NBCe1; Figure 4A; Gesek and Schoolwerth, 1991; Feraille et al., 1994; Ruiz et al., 1998). Recently, Nakamura et al. (2020) showed that, regarding sodium absorption in PT, insulin recruits an IRS (IRS2) different from the one orchestrating gluconeogenesis (IRS1). Insulin receptor substrate 2 acts through the AKT2/mTORC2 pathway (Nakamura et al., 2020; Figure 4A). In fact, mTORC2 is known to activate serum/glucocorticoid regulated kinase 1 (SGK1), that will then stimulate ENaC and NHE-3, increasing sodium reabsorption (Satoh et al., 2015). It has been suggested that, in healthy conditions, with rising insulin levels in fed state, IRS2 desensitize, suppressing sodium reabsorption at PT and increasing its delivery in the distal convoluted tubule (Figure 4B). Moreover, with IR, the desensitizing mechanism is abolished and therefore sodium will not reach the distal tubule (Ecelbarger, 2020; Figure 4C). Of notice, by recruiting distinct IRS, kidney cells can somehow dissociate pathways performing distinct functions.

Finally, it must be kept in mind that insulin can interact with intrarenal and systemic renin-angiotensin-aldosterone system in several ways (Muscogiuri et al., 2008) and therefore, indirectly interfere with sodium reabsorption in different mechanism out of the scope of this review.

### Albumin Absorption

The luminal membrane of PT cells is primarily responsible for the reabsorption of proteins that are freely filtered in the glomerulus by receptor-mediated endocytosis (Figure 4A; Christensen and Gburek, 2004). This is the case of albumin reabsorption that can have an important role in energy conservation. It has been suggested that albumin endocytosis is a regulated process, dependent on membrane receptors megalin and cubilin (Christensen and Birn, 2001). More recent evidence suggest that insulin might also have a role in the regulation of tubular albumin absorption (Kumari et al., 2019).

Albuminuria is of major clinical relevance in diagnosis and follow-up of kidney disease including subjects with diabetes. Insulin resistance was found to be associated with decreased INSR expression in tubular cells in rat models (Wang et al., 2005). In these observations, Kumari et al. (2019) analyzed urine samples from mice with targeted deletion of INSR from the renal PT. These mice had an impaired uptake of albumin, without any glomerulopathy. They also demonstrated that in healthy humans, albumin absorption capacity and excretion vary from the fast to the fed state. Moreover, IR was associated with microalbuminuria even in normoglycemia as described in the RISC study (Pilz et al., 2014) and thus can be present regardless of diabetes diagnosis. Altogether, these evidences suggest that albuminuria might be an important marker of kidney tubular dysfunction and might reflect tubule cells IR (Figure 4C). These reinforces the kidney contribution to diabetes development and highlights insulin and albumin dynamics prior and regardless of the development of diabetes.

## Kidney Insulin Clearance

In the systemic circulation, besides insulin metabolization by the liver, the kidney is the major site of insulin clearance (around 25%) (Elgee and Williams, 1954; Narahara et al., 1958); its action might be required to limit excessive insulin levels. Evidences supporting this theory started to rise in the middle of the 20th century (Zubrod et al., 1951; Elgee and Williams, 1954; Narahara et al., 1958; Ricketts and Wildberger, 1962). In a study from 1966, Beck et al. (1966) found that when insulin was injected intravenously in mice, it concentrated in liver and kidney; however, with higher insulin doses, raised insulin levels were found in rat’s kidney, while these levels were found to be reduced in liver. Nevertheless, this early study has some methodological limitations. Indeed, kidney insulin clearance remains constant in spite of insulinemia variations, but varies with creatinine clearance (Rubenstein et al., 1967). Globally, these evidences suggest that kidney insulin clearance is a non-saturable process, although dependent on glomerular filtration rate.

Insulin freely filtered in the glomerulus is absorbed by the lining cells of the PT (Figure 2). Upon entering the cell, insulin is transported through the luminal membrane into the PT cells and is degraded. Luminal insulin reabsorption limits urine insulin excretion, thus less than 2% of insulin reaches the urine in normal fasting conditions (Rubenstein and Spitz, 1968). Insulin is transported through the luminal membrane by a receptor-mediated endocytic mechanism (Rabkin et al., 1984). Endocytic internalization of insulin seems to be more related to insulin degradation than to insulin biological actions (Figure 2).

While glomerular filtration of insulin could not account for its total estimated renal extraction, a second mechanism was postulated. In this context, it was observed that a significant amount of insulin was cleared by the post-glomerular peritubular capillaries into the tubule cells (Chamberlain and Stimmler, 1967). In humans, this route represents around one-third of cleared insulin in the kidney, where it enters tubule cells not just by endocytosis, but also by INSR mediated uptake. In this case, through INSR binding, insulin signal to the kidneys’ tubular apparatus is crucial to maintain central physiologic functions, similarly to what happens in extra-renal tissues, namely regarding glucose homeostasis and blood pressure.

Insulin degrading activity has been observed at cytosol, lysosomes and mitochondria in addition to the membrane, indicating that it occurs in distinct cell sites. Degradation at the membrane level, however, seems to represent less than 2% of total degrading activity (Rabkin et al., 1984). Insulin can be initially hydrolyzed by an insulin protease followed by the action of plasma-membrane-associated or lysosomal proteases. This pathway can degrade insulin entering through both luminal and contraluminal membrane. In another possible pathway, endocytic vesicles containing insulin fuse with lysosomes. This pathway comprehends glutathione-insulin transhydrogenase (GIT) action, followed by hydrolysis of intact A and B chains by lysosomal proteases, and seems to need insulin internalization. It may act primarily on insulin delivered by luminal uptake and it is most active when supraphysiological levels of insulin are present (Rabkin et al., 1984).

Azizi et al. (2015) demonstrated that, in a culture of human adipose microvascular endothelial cells, insulin can go through microvascular endothelial cells by transcytosis (Azizi et al., 2015). Regarding insulin handling in the kidney tubule, Dahl et al. (1989) hypothesized that insulin molecules could also pass tubular cells by a retroendocytic pathway instead of being degraded. The authors demonstrated that cultured opossum kidney cells exhibited a retroendocytic pathway for insulin (Dahl et al., 1989). Using the same model, the authors later demonstrated that inhibition of insulin degradation diverted intact insulin from the degradative to the retroendocytic pathway (Dahl et al., 1990). Although captivating, especially regarding a potential contribution to hyperinsulinemic states, this hypothesis was not further explored.

Whether insulin clearance mechanisms attributed to other organs affects renal function and insulin clearance it is not clear. For example, the lack of CEACAM-1, a key protein enrolled in hepatic insulin clearance driving hyperinsulinemia, in the kidney leads to increased renin levels contributing to a potentiation of the RAS system and hypertension. These effects are exacerbated upon high fat diet exposure. Hence, the described CEACAM-1 renal effects can be due to the lack of its expression as well as the observed hyperinsulinemia (Huang et al., 2013; Li et al., 2015).

Despite early conflicting results, further studies showed that insulin is excreted in urine. However, in physiological conditions, it represents a minimal proportion of insulin filtered in the glomerulus. In health, a minor amount of insulin appears in the urine, as the majority is absorbed in PT. Tubule absorbing capacity of insulin does not saturate and thus the insulin fraction excreted in urine is constantly small, regardless of insulin levels. However, the amount of insulin excreted in urine varies physiologically (e.g., fasting and post-prandial) and in pathological conditions (obesity, diabetes) depending on the affected nephron region (e.g., glomerulopathy vs. tubulopathy) (Rubenstein and Spitz, 1968). Considering that insulin is internalized in the apical membrane by a receptor-mediated endocytic mechanism, the increased urinary insulin excretion might represent a tubular dysfunction. Subjects with tubulopathy show large amounts of insulin in urine approximating the amount that is filtered (Rabkin et al., 1984). Conversely, subjects with nephrotic syndrome show normal amounts of insulin in urine. When both glomerular and tubule lesion occur urine insulin excretion increase (Rabkin et al., 1984).

## Clinical Insights on Insulin Dysregulation

Insulin resistance is a common feature in chronic kidney disease (CKD) patients, even in absence of diabetes (DeFronzo et al., 1981; Shinohara et al., 2002; Becker et al., 2005; Kobayashi et al., 2005; Landau et al., 2011), and it is a risk factor for CKD progression (Fox et al., 2004). Its prevalence in CKD ranges from 30 to 50%, and this mainly depends on the adopted method of measurement (Spoto et al., 2016). Insulin resistance can be detected at the very early stages, when eGFR is still within the normal range, suggesting a potential role in triggering CKD (Fliser et al., 1998). A large study based on the Atherosclerosis Risk in Communities (ARICs) cohort confirmed that CKD development increases in strict parallelism with the number of metabolic syndrome criteria measured in non-diabetic adults, and this relationship remains significant even after controlling for the development of diabetes and hypertension (Kurella et al., 2005). Insulin resistance has also been associated with prevalent CKD and rapid decline in renal function in elderly, non-diabetic, Asian individuals (Cheng et al., 2012), and with microalbuminuria in the general population (Mykkänen et al., 1998), and in patients with T1DM (Yip et al., 1993; Ekstrand et al., 1998) and T2DM (Groop et al., 1993), indicating that this relationship is independent of diabetes (Mykkänen et al., 1998; Chen et al., 2003, 2004). The proposed mechanism by which IR contributes to kidney damage involves the worsening of renal hemodynamics through activation of the sympathetic nervous system (Rowe et al., 1981), sodium retention, decreased Na^+^, K^+^-ATPase activity, and increased GFR (Gluba et al., 2013).

The etiology of IR in CKD is multifactorial, depending on classical and CKD-peculiar risk factors, such as physical inactivity, inflammation and oxidative stress, adipokine derangements, vitamin D deficiency, metabolic acidosis, anemia and microbial toxins (Spoto et al., 2016).

Long-term hemodialysis has a positive effect on IR (DeFronzo et al., 1978), but there is little clinical data regarding the effect of peritoneal dialysis.

In addition to being a risk factor for CKD onset and progression, IR is also involved in the increased cardiovascular (CV) risk in this population. IR may be responsible for high blood pressure via direct stimulation of renin–angiotensin–aldosterone system (Nickenig et al., 1998), activation of sympathetic system (Sowers et al., 2001) and downregulation of the natriuretic peptide system (Sarzani et al., 1999).

However, the association between IR and CV complications in CKD patients is still to be clarified, as well as the relationship between IR and all-cause and CV mortality.

A positive association between IR and all-cause mortality was found in smokers and physically inactive CKD patients (Xu et al., 2014) and in a small cohort of 170 Japanese, non-diabetic, dialysis patients (Shinohara et al., 2002), whereas association with CV mortality was found in a cohort of peritoneal patients. However, no association was found in the ULSAM cohort, including 3-4 stage CKD patients (Jia et al., 2014) and in a small study performed in peritoneal dialysis patients (Sánchez-Villanueva et al., 2013).

Even though the prognostic value of IR for death and CV events need to be clarified, the association with CKD is well established. Even in this case, however, if IR is responsible of the onset and progression of CKD, or if CKD is responsible for IR is still to be clarified. A possible answer to this question could derive from clinical trials aiming at assessing the effect of drugs used for IR treatment on kidney function.

Thiazolidinediones (TZDs) are a class of oral diabetic medications that increase insulin sensitivity by acting on PPARγ (Yki-Järvinen, 2004). The effect of TZDs on kidney function has been previously described in mice models (Fujii et al., 1997). Treatment with TZDs has been demonstrated to improve insulin sensitivity in patients with T2DM after a 3-month treatment, and to reduce albuminuria, the last effect likely mediated by the concurrent increase in serum adiponectin concentration (Miyazaki et al., 2007). These results were confirmed in a meta-analysis of 15 double-blind, randomized, clinical trials involving diabetic patients (Sarafidis et al., 2010) and in a large study involving 4351 diabetic patients (Lachin et al., 2011). Even though a meta-analysis reported an increase in cardiovascular mortality linked to the use of TZDs in dialysis patients (Nissen and Wolski, 2007), no definitive proof are available on the risk related to this medication in this population.

Another interesting class of hypoglycemic drugs with positive kidney outcomes are the SGLT2 inhibitors (SGLT2i) which inhibit glucose and sodium reabsorption in the PT (Ferrannini, 2017). These drugs have a renoprotective effect in patients with T2DM (Perkovic et al., 2019) independently of glycemic control (Cherney et al., 2017). The renal protective effect can also be attributed to altered hemodynamics, reduced inflammation and fibrosis as well as controlled blood pressure and weight loss (Williams et al., 2020). In rats treated with SGLT2i the glycemic improvement was accompanied by a decrease in insulin and lipid levels (Huang et al., 2020). Moreover, the actions of SGLT2i are associated with increased insulin sensitivity and decreased albuminuria (Cherney et al., 2017). Interestingly, Jaikumkao et al. observed that in an animal model of diet induced obesity characterized by IR and impaired renal function dapagliflozin treatment resulted in improved IR, renal function and renal insulin signaling (Jaikumkao et al., 2018).

## Conclusion

Insulin is a hormone which acts not only on the most recognized insulin-responsive organs (liver, adipose tissue, and skeletal muscle), but also on the kidney. Moreover, the kidney has a primordial role in insulin clearance and may impact on insulin plasma levels. Whereas its main action is mainly related to homeostasis of glucose, including modulation of gluconeogenesis and lipolysis, in kidney the effects of insulin and IR change according to whether the target is in glomeruli or tubules. More specifically, if in glomerular podocytes insulin promotes glucose uptake, with an involvement in barrier permeability, in tubules it contributes to glucose reabsorption and gluconeogenesis regulation, and plays an important role in sodium homeostasis.

Importantly, insulin intervenes in albumin reabsorption at tubular level. Moreover, IR has been associated with microalbuminuria even in normoglycemia, and thus can be present regardless of diabetes diagnosis. These findings reinforce the kidney contribution to diabetes development and highlights insulin and albumin dynamics prior and regardless of the development of diabetes.

Insulin is cleared in the PT of kidney by two major routes, either by absorption of filtered insulin, or by post-glomerular capillary secretion, and only a minor amount appears to be excreted in urine. A decreased renal insulin clearance might lead to higher insulin levels, in the organ or systemically, favoring IR. Nonetheless, the impact of renal insulin clearance affection in the kidney or in insulin plasma levels still needs to be further unveiled.

It is clear now that kidney is not a mere target of insulin action, but insulin, more precisely IR, is also able to trigger CKD even in absence of diabetes. IR has been associated with prevalent CKD, rapid decline in renal function and microalbuminuria in the general population and in diabetic patients. In addition to being a risk factor for CKD onset and progression, IR is also involved in the increased cardiovascular risk in this population. However, if IR is responsible for the onset and progression of CKD, or if CKD is responsible for IR is still to be clarified. Preliminary confirmations come from clinical trials aiming at exploring the effect of TZDs, a class of oral diabetic medications, on kidney function. However, more focused studies, aiming also at testing the safety of these medications in CKD patients, are needed to better understand if treatment of IR may improve renal function in this population.

## Author Contributions

AP and MPM conceptualized the study. All authors originally drafted the manuscript, reviewed, edited, and critically approved the final version of the manuscript.

## Conflict of Interest

The authors declare that the research was conducted in the absence of any commercial or financial relationships that could be construed as a potential conflict of interest.

## Abbreviations

ADP: adenosine diphosphate
ATP: adenosine triphosphate
CEACAM-1: carcinoembryonic antigen-related cell adhesion molecule 1
cGMP: cyclic guanosine monophosphate
CKD: chronic kidney disease
CV: cardiovascular
DKD: diabetic kidney disease
DNL: *de novo* lipogenesis
eGFR: estimated glomerular filtration rate
ENaC: epithelial sodium channel
ER: endoplasmic reticulum
FFA: free fatty acid
FoxO1: forkhead box O1
GBM: glomerular basement membrane
GIT: glutathione-insulin transhydrogenase
GLUT1: glucose transporter 1
GLUT2: glucose transporter 2
GLUT4: glucose transporter 4
GRB2: growth factor receptor-bound protein 2
GSK3: glycogen synthase kinase 3
GSV: GLUT storage vesicle
IDE: insulin-degrading enzyme
INSR: insulin receptor
IR: insulin resistance
IRS1: insulin receptor substrate 1
IRS2: insulin receptor substrate 2
MAPK: mitogen activated protein kinase
mTOR: mammalian target of rapamycin
NAFLD: non-alcoholic fatty liver disease
NBCe1: sodium bicarbonate cotransporter 1
NHE-3: sodium/hydrogen exchanger 3
NKCC2: sodium-potassium chloride cotransporter-2
PGC1 α: peroxisome proliferator-activated receptor- γ coactivator 1 α
PI3K: phosphatidylinositol 3-kinase
PIP2: phosphatidylinositol 4,5 biphosphate
PIP3: phosphatidylinositol 3,4,5 triphosphate
PKB: protein kinase B
PKG: cGMP-dependent protein kinase G
PKGI: cGMP-dependent protein kinase G isoform I
PPAR γ: peroxisome proliferator-activated receptor γ
PT: proximal tubule
PTEN: phosphatase and tensin homolog
PTP1B: protein tyrosine-phosphatase 1B
SGK1: serum/glucocorticoid regulated kinase 1
SGLT: sodium-glucose transport proteins
SGLT2: sodium-glucose co-transporter 2
SGLT2i: sodium-glucose co-transporter-2 inhibitors
SHIP2: SH2-domain-containing inositol phosphatase 2
SREBP-1c: sterol regulatory element binding protein-1c
T1DM: type 1 diabetes mellitus
T2DM: type 2 diabetes mellitus
TZDs: thiazolidinediones
Vamp2: vesicle-associated membrane protein 2.
